# ﻿Morphology and multigene phylogeny reveal three new species of *Distoseptispora* (Distoseptisporales, Distoseptisporaceae) on palms (Arecaceae) from peatswamp areas in southern Thailand

**DOI:** 10.3897/mycokeys.102.112815

**Published:** 2024-02-09

**Authors:** Omid Karimi, K. W. Thilini Chethana, Antonio R. G. de Farias, Raheleh Asghari, Saithong Kaewchai, Kevin D. Hyde, Qirui Li

**Affiliations:** 1 State Key Laboratory of Functions and Applications of Medicinal Plants, Guizhou Medical University, Guiyang 550004, China; 2 School of Science, Mae Fah Luang University, Chiang Rai 57100, Thailand; 3 Center of Excellence in Fungal Research, Mae Fah Luang University, Chiang Rai 57100, Thailand; 4 Princess of Naradhiwas University, 99 Moo 8, Kok Kian, Muang District, Narathiwat Province, 9600 Thailand; 5 Mushroom Research Foundation, 128 M.3 Ban Pa Deng T. Pa Pae, A. Mae Taeng, Chiang Mai 50150, Thailand; 6 Innovative Institute for Plant Health, Zhongkai University of Agriculture and Engineering, Haizhu District, Guangzhou 510225, China

**Keywords:** asexual morph, molecular phylogeny, novel taxa, saprobic fungi, Sordariomycetes, taxonomy

## Abstract

Peatswamp forest is a unique habitat that supports high biodiversity, particularly fungal diversity. The current study collected submerged and dead plant parts from *Eleiodoxaconferta*, *Eugeissonatristis* and *Licualapaludosa* from a peatswamp forest in Narathiwat Province, Thailand. Morphological features coupled with multigene phylogenetic analyses of ITS, LSU, *rpb2* and *tef1-α* sequence data identified our isolates as new *Distoseptispora* species (viz. *D.arecacearum***sp. nov.**, *D.eleiodoxae***sp. nov.** and *D.narathiwatensis***sp. nov.**). Morphological descriptions, illustrations and notes are provided.

## ﻿Introduction

Most peatswamp forests can be found in tropical rainforests where peat is submerged for most of the year and characterised by low nutrient contents and high acidity due to lack of fully decomposed plant materials (Page et al. 1999, 2011; Jackson et al. 2009; Lampela et al. 2016; Ratnayake 2020). Peatswamp forests are unique ecosystems due to their high species diversity and significant role in maintaining a stable global climate. They function as carbon sinks, storing twice as much carbon as all global forest biomass (Hakim et al. 2017; Fujimoto et al. 2019; Shuhada et al. 2020). Beyond carbon storage, peatlands offer valuable benefits. They play vital roles in the water cycle, storing and filtering water and mitigating floods by slowing peak flows. Home to diverse plants and animals, these wetlands support millions of people. Additionally, they hold archaeological relics and provide insights into past environmental conditions through their peat layers, aiding predictions about the future climate (Parish et al. 2008; Posa et al. 2011; Minayeva and Sirin 2012; UNEP 2022). Asian peatlands are amongst the most diverse and geographically extensive in the world, with over 160 million hectares and the majority of tropical peatlands are found in Southeast Asia (e.g. Brunei, Indonesia, Malaysia, Papua New Guinea and Thailand) (UNEP 2022).

These habitats support many flora, including an extensive number of bryophytes, ferns and palms (Arecaceae) (Prentice 2011; Rieley 2016). Arecaceae comprises iconic monocotyledonous flowering plants belonging to 188 genera and 2,585 species that are distributed throughout tropical and subtropical areas of the world. However, they are most diverse in highly threatened moist tropical forest habitats (Dransfield et al. 2008; Palmweb 2023; POWO 2023). In peatswamp forests, many palm species, such as *Arecamacrocalyx* Zipp. ex Blume, *Calamusconcinnus* Mart, *Cyrtostachysrenda* Blume, *Eugeissonatristis* Griff, *Eleiodoxaconferta* (Griff.) Burret, *Licualalongicalycata* Furtado, *L.paludosa* Griff, *Metroxylonsagu* Rottb and *Nengapumila* (Blume) H.Wendl. ex Schaedtler can be found (Calabon et al. 2022), exerting different biological functions.

Several studies on palm fungi have focused on saprobic, endophytic and plant pathogenic life modes from different habitats worldwide (Hyde 1988; Taylor et al. 1999; Fröhlich and Hyde 2000; Fröhlich et al. 2000; Hyde et al. 2000; Taylor and Hyde 2003; Pilantanapak et al. 2005; Lumyong et al. 2009; Liu et al. 2010; Wikee et al. 2013; Konta et al. 2016a, 2016b, 2016c, 2017, 2020a, 2020b, 2020c, 2021a, 2021b, 2023; Chou et al. 2019; El Meleigi et al. 2019; Kinge et al. 2019; Marin-Felix et al. 2019; Chen et al. 2020; Mapook et al. 2020; Zhang et al. 2020; Tian et al. 2022). Even though many palm trees grow in peatswamp forests, there are few records of fungal studies in these environments, mostly reported from Thailand (Pinruan et al. 2002, 2004a, 2004b, 2004c, 2004d, 2007, 2008, 2010a, 2010b, 2014; Pinnoi et al. 2003a, 2003b, 2004, 2006, 2009, 2010; Voglmayr and Yule 2006; Sivichai and Boonyuen 2010; Boonyuen et al. 2012), of which many lack molecular data. Pinnoi et al. (2006) studied saprobic fungi on dead palm material in the Sirindhorn peatswamp forest, Narathiwat Province, Thailand and listed 462 ascomycetous and basidiomycetous taxa from various parts of palm materials (such as dry, damp and submerged palm materials), based on morphological identification and also recorded five sporidesmium-like taxa. Pinnoi et al. (2009) identified 88 fungal species from 212 collections of *Calamus* sp. in Thailand, with six records resembling sporidesmium-like taxa.

*Distoseptispora* K.D. Hyde, McKenzie & Maharachch belongs to Distoseptisporaceae, Distoseptisporales, Sordariomycetes, Ascomycota and comprises sporidesmium-like taxa (Wijayawardene et al. 2022). Su et al. (2016) proposed Distoseptisporaceae to accommodate sporidesmium-like taxa with *Distoseptispora* as the type genus and *D.fluminicola* McKenzie, Hong Y. Su, Z.L. Luo & K.D. Hyde as the type species. Subsequently, Luo et al. (2019) introduced Distoseptisporales to accommodate Distoseptisporaceae, based on multigene phylogenetic analyses of LSU, SSU, *rpb2* and *tef1-α* sequence data. *Distoseptispora* is characterised by short, septate, olivaceous to brown conidiophores. The conidiogenous cells are monoblastic and determinate, bearing acrogenous conidia that are brown, euseptate, distoseptate or muriform and cut off by cross walls at the basal cell with a basal scar (Yang et al. 2018). The genus exhibits morphology similar to *Sporidesmium*, but can be distinguished by having shorter conidiophores and darker conidia with pale round apexes (Su et al. 2016). To date, *Distoseptispora* comprises 65 species listed in the MycoBank database (https://www.mycobank.org/; Accessed in August 2023), with molecular data available for all reported species in the GenBank. The estimated divergence time for Distoseptisporaceae is approximately 44.21 million years ago (MYA), after the Tertiary–Cretaceous extinction event (Hyde et al. 2020), which could have created conducive conditions for *Distoseptispora* to thrive as a saprobe on various hosts (Phukhamsakda et al. 2022).

Peatswamp forests are unique, endangered ecosystems and their fungal biodiversity is little known. Therefore, in the current study, we aimed to study fungal species on different palm materials from peatswamp forests in Thailand, based on morphology and phylogeny. This study introduces three new species, *Distoseptisporaarecacearum*, *D.eleiodoxae* and *D.narathiwatensis*, associated with *Eleiodoxaconferta*, *Eugeissonatristis* and *Licualapaludosa* from a peatswamp forest in Narathiwat Province, Thailand, based on morphological characteristics coupled with multigene phylogenetic analyses (ITS, LSU, *rpb2* and *tef1-α*).

## ﻿Materials and methods

### ﻿Sample collection, morphological study and isolation

Decaying leaves of *Eleiodoxaconferta*, *Eugeissonatristis* and *Licualapaludosa* were collected from a peatswamp forest in Narathiwat Province, Thailand, in April 2022. Wet (submerged) and dry (aerial part) palm specimens were placed in plastic bags and brought to the laboratory. The submerged materials were kept moist and examined periodically for fungal fruiting structures and the dry materials were examined immediately or incubated in moisture chambers. Small pieces of the collected specimens were examined under a Leica EZ4 stereomicroscope and isolated into axenic culture using a single spore technique (Choi et al. 1999) in the Difco potato dextrose agar (PDA) media supplemented with Streptomycin 0.5 g/l. Germinating spores were transferred to new PDA and incubated at 25 ± 1 °C in dark conditions for two weeks. The micro-morphological characters were examined and photographed using a digital camera (Canon 600D, Japan) fitted to a compound microscope (Nikon ECLIPSE Ni, Japan) and the measurements were obtained using the Tarosoft (R) Image Frame Work programme version 0.9.7 (Tarosoft, Thailand). The ex-type living cultures were deposited at the Mae Fah Luang University Culture Collection (MFLUCC) and the herbarium specimens at the Mae Fah Luang University Herbarium (MFLU). The Facesoffungi (FoF) and Index Fungorum numbers were obtained, as explained in Jayasiri et al. (2015) and Index Fungorum (http://www.indexfungorum.org), respectively.

### ﻿DNA extraction, PCR amplification and sequencing

Genomic DNA was extracted from fresh fungal mycelia using the Biospin Fungus Genomic DNA Extraction Kit (BioFlux, P.R. China), according to the manufacturer’s standard protocol. Polymerase chain reactions (PCR) were conducted to amplify the internal transcribed spacer region rDNA (ITS), 28S large subunit rDNA (LSU), RNA polymerase II second largest subunit (*rpb2*) and translation elongation factor 1-alpha (*tef1-α*) using primers and conditions listed in Table 1. The PCR products were visualised on 1% agarose gels, stained with 4S Green Stain and sequenced at SolGent Co., Ltd (South Korea).

**Table 1.** Primers and PCR protocols.

Gene regions	Primers	PCR conditions	References
ITS	ITS5/ITS4	95 °C for 4 min, 40 cycles of 94 °C for 45 s, 56 °C for 1 min and 72 °C for 2 min, 72 °C for 10 min	White et al. (1990)
LSU	LR0R/LR5	94 °C for 3 min, 40 cycles of 94 °C for 30 s, 50 °C for 45 s and 72 °C for 2 min, 72 °C for 10 min	Vilgalys and Hester (1990), Rehner and Samuels (1994)
*rpb2*	fRPB2-5f/fRPB2-7cR	95 °C for 5 min, 35 cycles of 95 °C for 1 min, 55 °C for 1.25 min and 72 °C for 2 min, 72 °C for 10 min	Liu et al. (1999)
*tef1-α*	EF1-983F/EF1-2218R	94 °C for 3 min, 40 cycles of 94 °C for 30 s, 54 °C for 50 s and 72 °C for 2 min, 72 °C for 10 min	Rehner (2001)

### ﻿Sequence alignment and Phylogenetic analyses

The obtained sequences of ITS, LSU, *rpb2* and *tef1-α* were assembled using SeqMan software version 7.1.0 (DNASTAR Inc., WI) and subjected to BLASTn search against the GenBank nucleotide database at National Center for Biotechnology Information (NCBI) to identify closely-related sequences. Sequence data of related taxa were obtained from previous publications (Su et al. 2016; Yang et al. 2018, 2021; Crous et al. 2019; Hyde et al. 2019; Luo et al. 2019; Monkai et al. 2020; Phukhamsakda et al. 2020; Sun et al. 2020; Ma et al. 2022; Zhai et al. 2022; Zhang et al. 2022; Afshari et al. 2023) and downloaded from the GenBank database (Table 2). The sequences were aligned using MAFFT v.7 online web server (http://mafft.cbrc.jp/alignment/server/index.html, Katoh et al. 2019) under default settings and the alignments were trimmed in NGPhylogeny online web server (https://ngphylogeny.fr/workflows/wkmake/3a4ab1bef8e7ff3c, Lemoine et al. 2019). The sequence datasets were combined using SequenceMatrix software version 1.9 (Vaidya et al. 2011). The Maximum Likelihood (ML) phylogenetic analysis was run in the CIPRES Science Gateway platform (Miller et al. 2010), using RAxMLHPC2 on the XSEDE (v. 8.2.10) tool (Stamatakis 2014) under the GTRCAT substitution model and 1,000 non-parametric bootstrap replicates. For Bayesian Inference (BI) analysis, the optimal substitution model of each region was determined using jModelTest2 on the CIPRES Science Gateway under the Akaike Information Criterion (AIC) (Darriba et al. 2012). Bayesian analysis was performed using MrBayes v. 3.2.6 on XSEDE at the CIPRES Science Gateway with four simultaneous Markov Chain runs for 1,000,000 generations. The resulting trees were visualised in FigTree v. 1.4.0 (Rambaut 2012) and edited in Microsoft PowerPoint 2019 (Forethought, Inc., The United States).

**Table 2.** GenBank accession numbers used in the phylogenetic analyses.

Taxon	Identifier	GenBank accession number			
		ITS	LSU	*rpb2*	*tef1-α*
*Aquapteridosporaaquatica*	MFLUCC 17-2371*	NR172447	NG075413	–	–
*A.fusiformis*	MFLU 18-1601*	MK828652	MK849798	–	MN194056
*Distoseptisporaadscendens*	HKUCC 10820	–	DQ408561	DQ435092	–
*D.amniculi*	MFLUCC 17-2129*	MZ868770	MZ868761	MZ892982	–
*D.appendiculata*	MFLUCC 18-0259*	MN163009	MN163023	–	MN174866
*D.aqualignicola*	KUNCC 21-10729*	OK341186	ON400845	OP413474	OP413480
*D.aquamyces*	KUNCC 21-10732*	OK341187	OK341199	OP413476	OP413482
*D.aquatica*	MFLUCC 15-0374*	MF077552	KU376268	–	–
*D.aquatica*	MFLUCC 16-0904	MK828649	MK849794	–	MN194053
*D.aquatica*	MFLUCC 18-0646	MK828648	MK849793	–	MN194052
*D.aquatica*	S-965	MK828647	MK849792	MN124537	MN194051
*D.aquisubtropica*	GZCC 22-0075*	ON527933	ON527941	ON533685	ON533677
***D.arecacearum***	MFLUCC 23-0211*	**OR234707**	**OR510857**	**OR250439**	**OR250442**
***D.arecacearum***	**MFLUCC 23-0212**	**OR354399**	**OR510860**	**OR481048**	**OR481045**
*D.atroviridis*	GZCC 20-0511*	MZ868772	MZ868763	MZ892984	MZ892978
*D.atroviridis*	GZCC 19-0531	MW133915	MZ227223	–	–
*D.bambusae*	MFLUCC 20-0091*	NR170068	NG074430	MT232881	MT232880
*D.bambusae*	MFLU 17-1653	MT232712	MT232717	MT232882	–
*D.bangkokensis*	MFLUCC 18-0262*	MZ518205	MZ518206	–	–
*D.cangshanensis*	MFLUCC 16-0970*	MG979754	MG979761	–	MG988419
*D.caricis*	CPC: 36498*	NR166325	MN567632	MN556805	–
*D.caricis*	CPC: 36442	MN562125	–	MN556806	–
*D.chinensis*	GZCC 21-0665	MZ474871	MZ474867	–	MZ501609
*D.clematidis*	MFLUCC 17-2145*	MT310661	MT214617	MT394721	–
*D.clematidis*	KUN-HKAS:112708	MW723056	MW879523	–	–
*D.crassispora*	KUMCC 21-10726*	OK310698	OK341196	OP413473	OP413479
*D.curvularia*	KUMCC 21-10725*	OK310697	OK341195	OP413472	OP413478
*D.cylindricospora*	KUN-HKAS:115796*	OK491122	OK513523	–	OK524220
*D.dehongensis*	KUMCC 18-0090*	MK085061	MK079662	–	MK087659
*D.dipterocarpi*	MFLUCC 22-0104 *	OP600053	OP600052	OP595140	–
*D.effusa*	GZCC 19-0532*	MW133916	MZ227224	–	–
***D.eleiodoxae***	MFLUCC 23-0213*	**OR234706**	**OR510856**	**OR250438**	**OR250441**
***D.eleiodoxae***	**MFLUCC 23-0214**	**OR354398**	**OR510859**	**OR481047**	**OR481044**
*D.euseptata*	MFLUCC 20-0154*	MW081539	MW081544	MW151860	–
*D.euseptata*	MFLU 20-0568	MW081540	MW081545	MW084996	MW084994
*D.fasciculata*	KUMCC 19-0081*	NR172452	NG075417	–	MW396656
*D.fluminicola*	MFLUCC 15-0417*	MF077553	KU376270	–	–
*D.fusiformis*	GZCC 20-0512*	MZ868773	MZ868764	MZ892985	MZ892979
*D.guizhouensis*	GZCC 21-0666*	MZ474868	MZ474869	MZ501611	MZ501610
*D.guttulata*	MFLUCC 16-0183*	MF077543	MF077554	–	MF135651
*D.hyalina*	MFLUCC 17-2128*	MZ868769	MZ868760	MZ892981	MZ892976
*D.hydei*	MFLUCC 20-0115*	MT734661	MT742830	MT767128	–
*D.lancangjiangensis*	DLUCC 1864*	MW723055	MW879522	–	–
*D.leonensis*	HKUCC 10822	–	DQ408566	DQ435089	–
*D.licualae*	MFLUCC 14-1163*	ON650686	ON650675	–	ON734007
*D.lignicola*	MFLUCC 18-0198*	MK828651	MK849797	–	–
*D.longispora*	HFJAU 0705*	MH555359	MH555357	–	–
*D.martinii*	CGMCC 3.18651	KU999975	KX033566	–	–
*D.meilingensis*	JAUCC 4728	OK562391	OK562397	–	OK562409
*D.mengsongensis*	HJAUP C2126*	OP787876	OP787874	–	OP961937
*D.multiseptata*	MFLUCC 15-0609*	KX710145	KX710140	–	MF135659
*D.nabanheensis*	HJAUP C2003*	OP787877	OP787873	–	OP961935
***D.narathiwatensis***	MFLUCC 23-0215*	**OR234708**	**OR510858**	**OR250440**	**OR250443**
***D.narathiwatensis***	**MFLUCC 23-0216**	**OR354400**	**OR510861**	**OR481049**	**OR481046**
*D.neorostrata*	MFLUCC 18-0376*	MN163008	MN163017	–	–
*D.nonrostrata*	KUNCC 21-10730*	OK310699	OK341198	OP413475	OP413481
*D.obclavata*	MFLUCC 18-0329*	MN163012	MN163010	–	–
*D.obpyriformis*	MFLUCC 17-1694*	–	MG979764	MG988415	MG988422
*D.obpyriformis*	DLUCC 0867	MG979757	MG979765	MG988416	MG988423
*D.pachyconidia*	KUMCC 21-10724*	OK310696	OK341194	OP413471	OP413477
*D.palmarum*	MFLUCC 18-1446*	MK085062	MK079663	MK087670	MK087660
*D.palmarum*	MFLU 18-0588	NR165897	NG067856	–	–
*D.phangngaensis*	MFLUCC 16-0857*	NR166230	–	–	MF135653
*D.rayongensis*	MFLUCC 18-0415*	NR171938	NG073624	–	MH463253
*D.rayongensis*	MFLU 18-1045	MH457172	MH457137	MH463255	–
*D.rostrata*	MFLUCC 16-0969*	MG979758	MG979766	MG988417	MG988424
*D.rostrata*	DLUCC 0885	MG979759	MG979767	–	MG988425
*D.rostrata*	MFLU 18-0479	NR157552	NG064513	–	–
*D.saprophytica*	MFLUCC 18-1238*	NR172454	NG075419	MW504069	MW396651
*D.septata*	GZCC 22-0078*	ON527939	ON527947	ON533690	ON533683
*D.sinensis*	HJAUP C2044*	OP787878	OP787875	–	OP961936
*D.songkhlaensis*	MFLUCC 18-1234*	MW286482	MW287755	–	MW396642
*D.submersa*	MFLUCC 16-0946	MG979760	MG979768	MG988418	MG988426
*D.suoluoensis*	MFLUCC 17-0224*	NR168764	NG068552	–	MF135654
*D.tectonae*	MFLUCC 12-0291*	KX751711	KX751713	KX751708	KX751710
*D.tectonigena*	MFLUCC 12-0292*	NR154018	KX751714	KX751709	–
*D.thailandica*	MFLUCC 16-0270*	MH275060	MH260292	–	MH412767
*D.thysanolaenae*	KUN-HKAS: 112710	MW723057	MW879524	–	MW729783
*D.thysanolaenae*	KUMCC 18-0182	MK045851	MK064091	–	MK086031
*D.tropica*	GZCC 22-0076*	ON527935	ON527943	ON533687	ON533679
*D.verrucosa*	GZCC 20-0434*	MZ868771	MZ868762	MZ892983	MZ892977
*D.wuzhishanensis*	GZCC 22-0077*	ON527938	ON527946	–	ON533682
*D.xishuangbannaensis*	KUMCC 17-0290*	MH275061	MH260293	MH412754	MH412768
*D.yongxiuensis*	JAUCC 4725	OK562388	OK562394	–	OK562406
*D.yongxiuensis*	JAUCC 4726	OK562389	OK562395	–	OK562407
*D.yunjushanensis*	JAUCC 4723	OK562392	OK562398	–	OK562411
*D.yunjushanensis*	JAUCC 4724	OK562393	OK562399	–	OK562410
*D.yunnanensis*	MFLUCC 20-0153*	MW081541	MW081546	MW151861	MW081541

Ex-type strains are indicated with an asterisk (*) after the collection number; “–” indicates unavailable sequences; sequences produced in the current study are in bold.

### ﻿Abbreviations

**CGMCC**: China General Microbiological Culture Collection Center, Chinese Academy of Sciences, Beijing, China; **CPC**: Collection of P.W. Crous, Utrecht, The Netherlands; **DLUCC**: Dali University Culture Collection, Yunnan, China; **GZCC**: Guizhou Culture Collection, Gui Yang, China; **HFJAU**: Herbarium of Fungi, Jiangxi Agricultural University, Nanchang, China; **HKUCC**: The University of Hong Kong Culture Collection, Hong Kong, China; **JAUCC**: Jiangxi Agricultural University Culture Collection, Nanchang, China; **KUMCC**: Kunming Institute of Botany Culture Collection, Kunming, China; **KUN-HKAS**: Herbarium of Cryptogams, Kunming Institute of Botany Academia Sinica; **MFLU**: Mae Fah Luang University Herbarium, Chiang Rai, Thailand; **MFLUCC**: Mae Fah Luang University Culture Collection, Chiang Rai, Thailand.

## ﻿Results

### ﻿Phylogenetic analyses

The combined ITS, LSU, *rpb2* and *tef1-α* dataset consisted of 83 strains, with *Aquapteridosporaaquatica* X.D. Yu, W. Dong & H. Zhang (MFLUCC 17-2371) and *A.fusiformis* Z.L. Luo, D.F. Bao, H.Y. Su & K.D. Hyde (MFLU 18-1601) as outgroup taxa (Table 2). The final alignment comprised 3,383 characters (ITS: 567 bp, LSU: 855 bp, *rpb2*: 1,051 bp, *tef1-α*: 909 bp), including gaps. The final ML optimisation likelihood value of the best RAxML tree was -33894.57 and the matrix had 1,637 distinct alignment patterns, with 29.85% undetermined characters or gaps. Estimated base frequencies were as follows: A = 0.240315, C = 0.262752, G = 0.283175, T = 0.213758; substitution rates AC = 1.324179, AG = 3.427671, AT = 1.239665, CG = 0.914354, CT = 6.950002, GT = 1.0; gamma distribution shape parameter α = 0.273123. The RAxML and Bayesian analyses yielded a similar tree topology.

**Figure 1.**
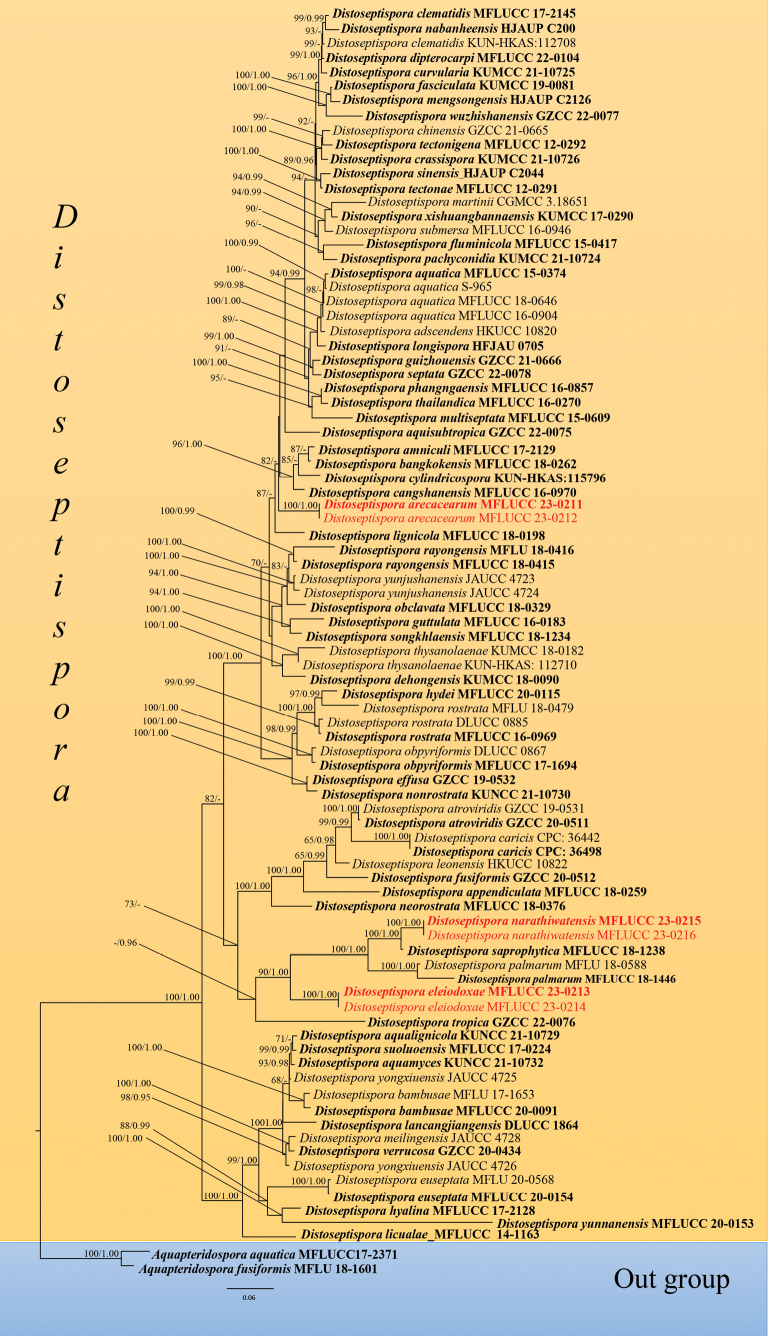
Maximum Likelihood tree generated from combined ITS, LSU, *rpb2* and *tef1-α* sequence data. Bootstrap support values ≥ 65% and Bayesian posterior probabilities ≥ 0.95 are demonstrated at the nodes. The new taxa are indicated in red bold. Ex-type strains are in black bold.

The topology of our phylogenetic tree is nearly identical to previous publications, but there are some differences, which may be due to different taxon sampling. As new species are introduced into this genus frequently, taxon sampling conducted for different studies varies. In our phylogenetic analyses, two strains of the new species *Distoseptisporaarecacearum* (MFLUCC 23-0211 and MFLUCC 23-0212) formed a robust subclade (100% ML, 1.00 PP) independently. The species has close relationships with *D.amniculi* (MFLUCC 17-2129), *D.bangkokensis* (MFLUCC 18-0262), *D.cangshanensis* (MFLUCC 16-0970) and *D.cylindricospora* (KUN-HKAS:115796) with 82% ML bootstrap support. The other two new species, *D.eleiodoxae* and *D.narathiwatensis*, clustered with *D.saprophytica* (MFLUCC 18-1238), *D.palmarum* (MFLU 18-0588 and MFLUCC 18-1446) and *D.tropica* (GZCC 22-0076) with 0.96 PP support. *Distoseptisporaeleiodoxae* (strains MFLUCC 23-0213 and MFLUCC 23-0214) formed a robust subclade (100% ML, 1.00 PP) basal to *D.narathiwatensis* (MFLUCC 23-0215 and MFLUCC 23-0216), *D.saprophytica* (MFLUCC 18-1238) and *D.palmarum* (MFLU 18-0588 and MFLUCC 18-1446) with 90% ML and 1.00 PP support. *Distoseptisporanarathiwatensis* (MFLUCC 23-0215 and MFLUCC 23-0216) formed a sister clade with *D.saprophytica* (MFLUCC 18-1238) with 100% ML and 1.00 PP support (Fig. 1).

### ﻿Taxonomy

#### ﻿ Distoseptispora arecacearum

Taxon classificationFungiDistoseptisporalesDistoseptisporaceae

O. Karimi, Q.R. Li & K.D. Hyde sp. nov.

9324C5D1-2128-5124-A0AB-01614294919F

Index Fungorum number: IF900843

Facesoffungi number: FoF14756

Fig. 2

##### Etymology.

The epithet ‘‘*arecacearum*’’ refers to host family, Aceraceae.

##### Holotype.

MFLU 23-0276.

##### Description.

***Saprobic*** on submerged rachis of *Licualapaludosa* in peatswamp forest. ***Sexual morph***: Undetermined. ***Asexual morph***: Hyphomycetous. Colonies gregarious or scattered, effuse, hairy, dark brown to black. ***Mycelium*** mostly immersed, composed of branched, septate, smooth hyphae. ***Conidiophores*** 70–140 × 5.1–6.3 µm (x̄ = 110 × 5.5 µm, n = 20), macronematous, mononematous, unbranched, erect, straight or flexuous, cylindrical, smooth, thick-walled, brown, 4–7 septa, sometimes consists a swollen cell in the middle or towards the apex. ***Conidiogenous cells*** 13–25 × 4.5–6 µm (x̄ = 17 × 5 µm, n = 20), monoblastic or polyblastic, terminal or subterminal, determinate, cylindrical, brown. ***Conidia*** 25–60 × 7–17 µm (x̄ = 44 × 10 µm, n = 30), acrogenous, solitary, cylindrical, obclavate to obpyriform or irregular, straight or curved, 4–10-distoseptate, brown, thick-walled, smooth, round apex, truncated base, sometimes with percurrent regeneration forming a secondary conidium from the conidial apex.

**Figure 2.**
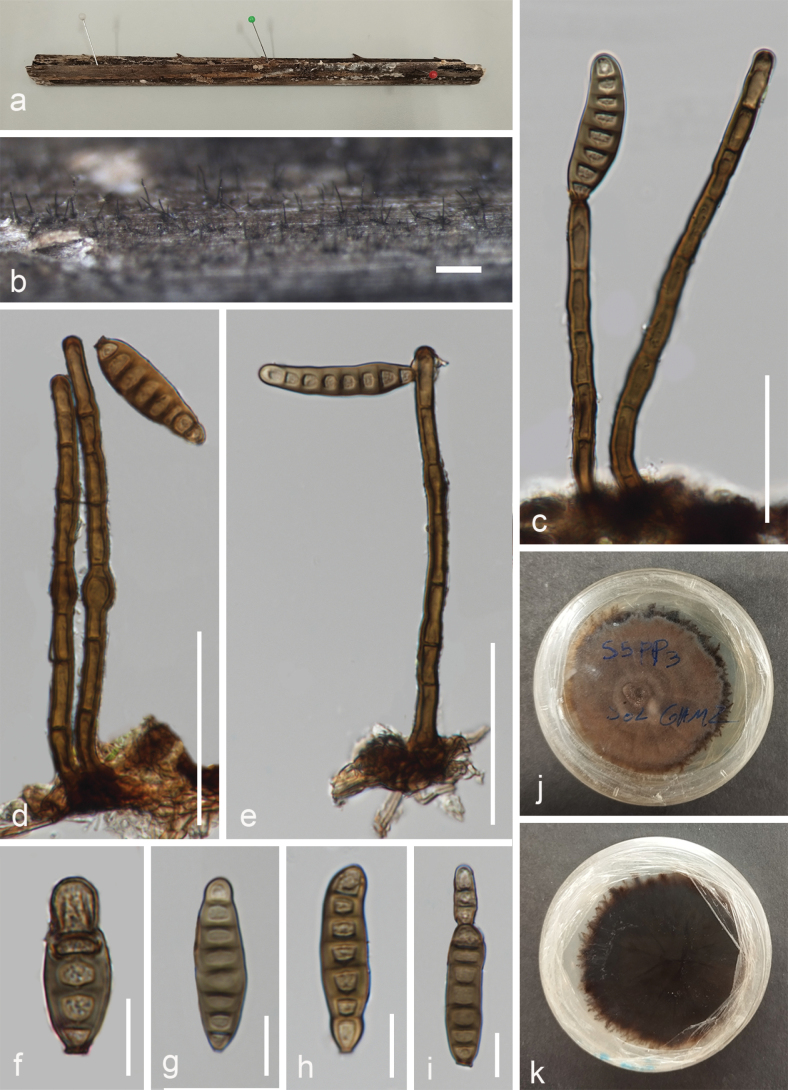
*Distoseptisporaarecacearum* (MFLU 23-0276, holotype) **a** host material **b** colonies on the substrate **c–e** conidiophores and conidia **f–i** conidia **j, k** culture on PDA. Scale bars: 200 µm (**b**); 50 µm (**c–e**); 10 µm (**f–i**).

##### Culture characteristics.

Colonies grown on PDA, reaching 50 mm in diameter after 15 days at 25 °C, under dark conditions, circular, fimbriate edge, flat, dull surface, radiating outwards, felted, medium dense, without pigment diffusion and sporulation, brown on the top, reverse dark brown to black.

##### Material examined.

Thailand. Narathiwat Province: Yi-ngo District, peatswamp forest, on submerged rachis of *Licualapaludosa*, 06 April 2022, Omid Karimi, S5PP3SG (MFLU 23-0276, ***holotype***); ex-type culture MFLUCC 23-0211, additional living culture MFLUCC 23-0212.

##### Notes.

Morphologically, our proposed new species is similar to *Distoseptisporadehongensis* W. Dong, H. Zhang & K.D. Hyde and *D.obpyriformis* Z.L. Luo & H.Y. Su in having macronematous, mononematous, unbranched, erect, straight or flexuous, cylindrical, septate conidiophores, terminal, determinate, cylindrical, brown conidiogenous cells and acrogenous, distoseptate, straight or curved conidia (Luo et al. 2018; Hyde et al. 2019). However, our isolate differs from *D.dehongensis* (HKAS 101738) in having longer and wider conidiophores (70–140 × 5.1–6.3 µm vs. 45–80 × 4–5 µm), with swollen cells, longer and wider conidia (25–60 × 7–17 µm vs. 17–30 × 7.5–10 µm) and more distosepta (4–10-distoseptate vs. 3–5-distoseptate). *Distoseptisporaarecacearum* (MFLU 23-0276) differs from *D.obpyriformis* (MFLU 18–0476) in having conidiophores with swollen cells and shorter conidia (25–60 µm vs. 53–71 µm) (Luo et al. 2018). The BLASTn searches of the ITS sequence of *D.arecacearum* (MFLUCC 23-0211) resulted in *D.aquatica* Z.L. Luo, H.Y. Su & K.D. Hyde (MFLUCC 18-0646) with 92.21% similarity across 100% of the query sequence coverage, while the LSU sequence of *D.arecacearum* has 99.09% similarity across 100% of the sequence coverage with *D.phangngaensis* J. Yang, Maharachch. & K.D. Hyde (MFLUCC 16-0857). *Distoseptisporaarecacearum* (MFLU 23-0276) is easily distinguishable from *D.aquatica* (HKAS 83991) in having longer conidiophores (70–140 µm vs. 29–41 μm) and shorter conidia (25–60 µm vs. 110–157 µm) with less distosepta (4–10-distoseptate vs. 15–28-distoseptate) (Su et al. 2016). *Distoseptisporaarecacearum* (MFLU 23-0276) differs from *D.phangngaensis* (MFLU 17-0855) in having longer conidiophores (70–140 µm vs. 18–30(–40) μm) and shorter conidia (25–60 µm vs. 165–350 µm) (Yang et al. 2018). Therefore, we introduced *D.arecacearum* (MFLU 23-0276) as a novel species, based on morphological and phylogenetic analyses.

#### ﻿ Distoseptispora eleiodoxae

Taxon classificationFungiDistoseptisporalesDistoseptisporaceae

O. Karimi, Q.R. Li & K.D. Hyde sp. nov.

802F00CB-4010-51D6-8A16-EC7A3F3E7F84

Index Fungorum number: IF900844

Facesoffungi number: FoF14757

Fig. 3

##### Etymology.

The epithet “*eleiodoxae*” refers to the name of the host genus, *Eleiodoxaconferta*.

##### Holotype.

MFLU 23-0277.

##### Description.

***Saprobic*** on submerged rachis of *Eleiodoxaconferta* in peatswamp forest. ***Sexual morph***: Undetermined. ***Asexual morph***: Hyphomycetous. ***Mycelium*** immersed to superfacial, septate, smooth, brown to dark brown. ***Colonies*** on submerged rachis, solitary, scattered, dark brown to black. ***Conidiophores*** 71–161 × 5–6.5 µm (x̄ = 110 × 5.7 µm, n = 20), macronematous, mononematous, cylindrical, erect, straight to flexuous, unbranched, smooth or finely verrucose, thick-walled, dark brown, 5–10-septate with lobed basal cells, percurrent proliferations at the apex. ***Conidiogenous cells*** 13.5–18.8 × 5–6.8 µm (x̄ = 15.96 × 5.6 µm, n = 20), holoblastic, monoblastic, terminal, integrated, cylindrical to ampulliform, percurrent, brown to dark brown, smooth. ***Conidia*** 31.5–48 × 13.5–15.8 µm (x̄ = 40.8 × 14.8 µm, n = 30), secession schizolytic, solitary, obpyriform, rostrate, truncated base, 6–7-euseptate, verrucose, thick-walled, brown with dark brown to black cells in the middle, paler towards the apex.

**Figure 3.**
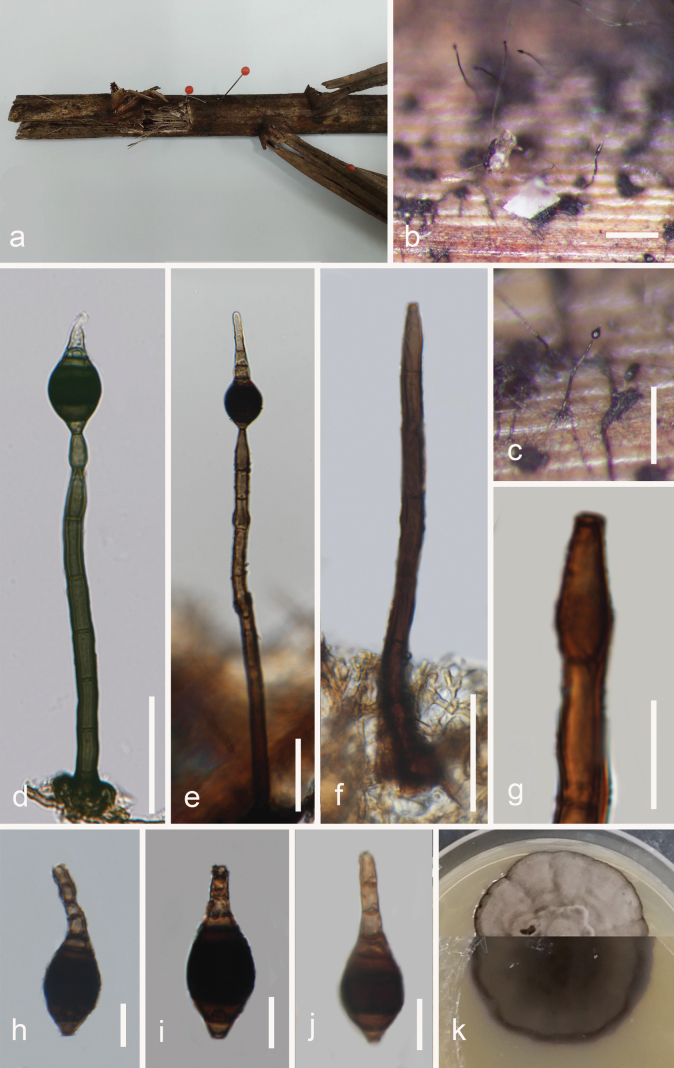
*Distoseptisporaeleiodoxae* (MFLU 23-0277, holotype) **a** host material **b**, **c** colonies on the substrate **d–f** conidiophores and conidia **g** conidiogenous cell **h–j** conidia **k** culture on PDA (top and reverse). Scale bars: 100 µm (**b, c**); 30 µm (**d–f**); 10 µm (**g–j**).

##### Culture characteristics.

Colonies grown on PDA, reaching 30 mm in diameter after 15 days at 25 °C, under dark conditions, circular, entire to radially with lobate edge, well-defined margin, low convex, dull surface, felted, dense, mycelium superficial to immersed, without pigment diffusion and sporulation, greyish-brown on the top with dark brown margin, reverse brown with dark brown centre and margin.

##### Material examined.

Thailand. Narathiwat Province: Yi-ngo District, peatswamp forest, on submerged rachis of *Eleiodoxaconferta*, 06 April 2022, Omid Karimi, S5PP8N1SG (MFLU 23-0277, ***holotype***); ex-type culture MFLUCC 23-0213, additional living culture MFLUCC 23-0214.

##### Notes.

*Distoseptisporaeleiodoxae* (MFLU 23-0277) shares similar characteristics with *D.tropica* J. Ma & Y.Z. Lu (HKAS 123761), in having macronematous, mononematous, cylindrical, erect, straight, unbranched conidiophores with holoblastic, monoblastic, terminal, cylindrical, thick-walled conidiogenous cells and verrucose, rostrate conidia (Ma et al. 2022). However, *D.eleiodoxae* (MFLU 23-0277) differs from *D.tropica* (HKAS 123761) in having shorter and wider obpyriform conidia (31.5–48 × 13.5–15.8 µm vs. 39–75 × 7.5–10.5 µm), with broad and darker middle cells, no guttules and lacking conspicuous hyphae attachment to conidia. The BLAST search against GenBank showed that the ITS and LSU sequences of the new isolate, *D.eleiodoxae* (MFLUCC 23-0213), share 84.25% similarity across 100% sequence coverage with *D.tropica* (GZCC 22-0076) and 96.09% similarity across 100% sequence coverage with *D.effusa* L.L. Liu & Z.Y. Liu, respectively. *Distoseptisporaeleiodoxae* (MFLU 23-0277) differs from *D.effusa* (GZAAS 20-0427) in having shorter conidia (31.5–48 vs. 35.5–113 µm) (Yang et al. 2021). Based on a pairwise comparison of ITS, LSU, *rpb2* and *tef1-α* nucleotides, *D.eleiodoxae* (MFLUCC 23-0213) differs from *D.tropica* (GZCC 22-0076) in 70/536 bp (13.05%) for ITS, 50/834 bp (5.99%) for LSU, 141/1052 bp (13.40%) for *rpb2* and 96/888 bp (10.8%) for *tef1-α* (without including gaps). Therefore, we introduced *D.eleiodoxae* (MFLU 23-0277) as a novel species, based on the morphological evidence and according to the species delimitation guidelines proposed by Chethana et al. (2021) and Maharachchikumbura et al. (2021).

#### ﻿ Distoseptispora narathiwatensis

Taxon classificationFungiDistoseptisporalesDistoseptisporaceae

O. Karimi, Q.R. Li & K.D. Hyde sp. nov.

465DE60E-9E79-5FCF-8E34-0C0BF6A4D4F6

Index Fungorum number: IF900845

Facesoffungi number: FoF14758

Fig. 4

##### Etymology.

The epithet “*narathiwatensis*” refers to Narathiwat Province, where the holotype was collected.

##### Holotype.

MFLU 23-0278.

##### Description.

***Saprobic*** on dead petiole of *Eugeissonatristis* in peatswamp forest. ***Sexual morph***: Undetermined. ***Asexual morph***: Hyphomycetous. ***Colonies*** superficial, effuse, hairy, gregarious, brown. ***Mycelium*** immersed to superficial, composed of septate, branched, pale brown hyphae. ***Conidiophores*** 27–155 × 3–6.5(–7) μm (x̄ = 104 × 5 μm, n = 50), macronematous, mononematous, cylindrical, straight or flexuous, occasionally slightly curved in the middle and near the base and the apex, up to 10 septa, slightly constricted at septa, unbranched, brown, thin-walled, smooth, often containing inflated or constricted cells at the apex or middle, sometimes percurrent with annellations. ***Conidiogenous*** cells 7–17 × 4–5.5 μm (x̄ = 12.5 × 5 μm, n = 30), holoblastic, mono- to polyblastic, integrated, determinate, terminal and intercalary, subcylindrical, brown, smooth. ***Conidia*** 12–38 × 4.5–8 μm (x̄ = 27 × 6.5 μm, n = 30), secession schizolytic, solitary or occasionally catenate, dry, thin-walled, smooth, subcylindrical to obclavate to conical, straight or curved, 1–7-distoseptate, slightly constricted at septa, olivaceous to brown, apex rounded, truncated base with slightly pigmented scar, often the primary cells of conidia are narrower than the second ones which are often inflated.

**Figure 4.**
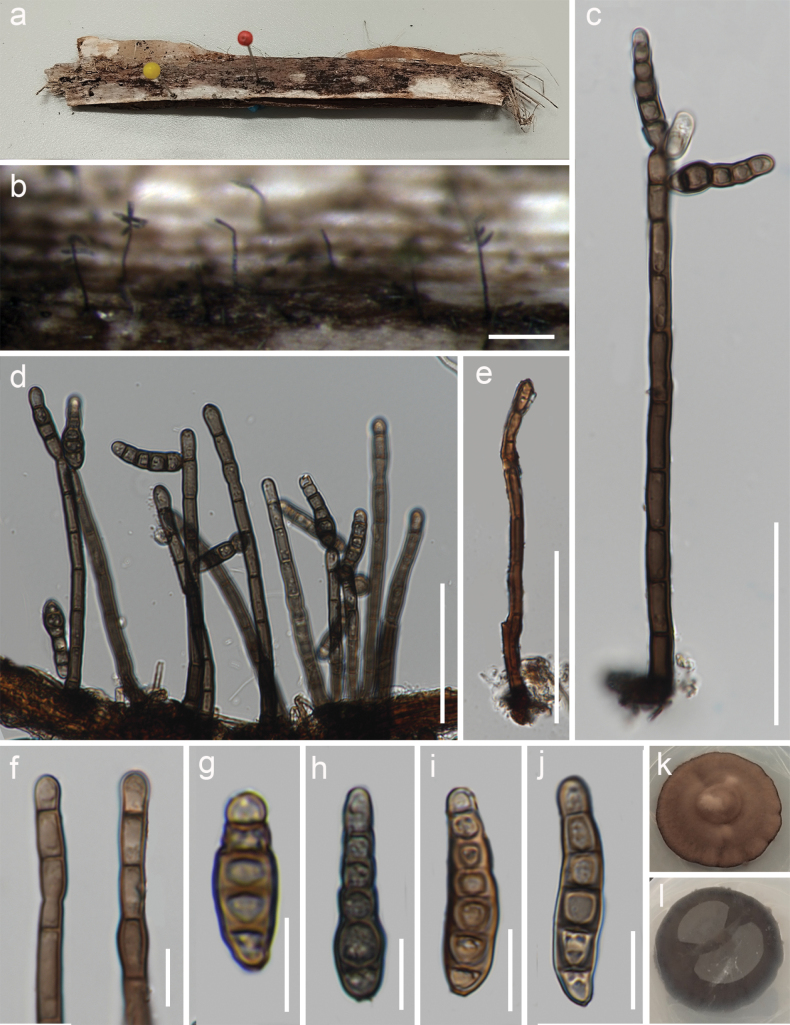
*Distoseptisporanarathiwatensis* (MFLU 23-0278, holotype) **a** host material **b** colonies on the substrate **c–e** conidiophores and conidia **f** conidiogenous cell **g–j** conidia **k, l** culture on PDA. Scale bars: 100 μm (**b**); 50 μm (**c–e**); 10 μm (**f–j**).

##### Culture characteristics.

Colonies grown on PDA, reaching 50 mm in diameter after 15 days at 25 °C, under dark conditions, circular, entire margin, well-defined margin, low convex, dull surface, felted, dense, mycelium mostly superficial, without pigment diffusion and sporulation, medium brown to reddish-brown with dark brown edge on the top, reverse-side dark brown to black.

##### Material examined.

Thailand. Narathiwat Province: Yi-ngo District, peatswamp forest, on dead petiole of *Eugeissonatristis*, 06 April 22, Omid Karimi, 35Y (MFLU 23-0278, ***holotype***); ex-type culture MFLUCC 23-0215, additional living culture MFLUCC 23-0216.

##### Notes.

*Distoseptisporanarathiwatensis* (MFLU 23-0278) is similar to *D.saprophytica* (MFLU 18-1568), but it can be distinguished in having longer and wider conidiophores (27–155 × 3–6.5 (–7) μm vs. 50–140 × 3.2–4.2 μm) and conidiogenous cells (7–17 × 4–5.5 μm vs. 5–11.5 × 3–4.5 μm). In *D.narathiwatensis* (MFLU 23-0278), the conidiophore is slightly curved at the base, middle and near the top in contrast to *D.saprophytica* (MFLU 23-0278), which is characterised by sharp curving near the base; also in *D.narathiwatensis*, the conidiophore cells are often inflated or constricted at the apex or middle which is not observed in *D.saprophytica* (Dong et al. 2021). Conidiogenous cells of *D.narathiwatensis* are terminal and intercalary and their conidia are not acrogenous as in *D.saprophytica*. The primary cell in the conidium is often narrower than the second one and the second cell is often inflated, which is not observed in *D.saprophytica*. The BLAST search against the GenBank showed that the ITS and *rpb2* sequences of the new isolate, *D.narathiwatensis* (MFLUCC 23-0215), share 98.33% similarity across 100% sequence coverage and 98.63% similarity across 78% sequence coverage with *D.saprophytica* (MFLUCC 18-1238), respectively. In a BLAST search against GenBank, the LSU and *tef1-α* sequences of *D.narathiwatensis* (MFLUCC 23-0215) share 99.3% similarity across 85% sequence coverage and 94.12% similarity across 94% sequence coverage with *D.palmarum* (MFLU 18-0588), respectively. However, *D.palmarum* is distinguished in having longer (12–38 μm vs. 35–180 μm), elongated, greenish-black to brown conidia (Hyde et al. 2019). Based on a pairwise comparison of ITS and LSU nucleotides, *D.narathiwatensis* (MFLUCC 23-0215) differs from *D.saprophytica* (MFLUCC 18-1238) by 22/580 bp (3.8%), 16/870 bp (1.8%) differences, respectively (without including gaps). Therefore, we introduced *D.narathiwatensis* (MFLU 23-0278) as a novel species, based on the morphological evidence and according to the species delimitation guidelines proposed by Chethana et al. (2021) and Maharachchikumbura et al. (2021).

## ﻿Discussion

Peatswamp forests are unique habitats found in only a few regions worldwide (Jackson et al. 2009). The destruction caused by humans threatens them; hence more extensive studies on fungal identification are needed before the extinction of fungal species. Pinnoi et al. (2006, 2009) recorded sporidesmium-like taxa on the palm species *Eleiodoxaconferta* and *Calamus* sp. in Sirindhorn peatswamp forest, Narathiwat, Thailand, based on morphological data. In this study, three new *Distoseptispora* species (*D.arecacearum*, *D.eleiodoxae and D.narathiwatensis*) from peatswamp forest in Thailand are introduced, based on multilocus phylogenetic analysis (ITS, LSU, *rpb2* and, *tef1-α*) (Fig. 1) and morphology (Figs 2–4).

The fungal diversity in peatswamp forest has not been well studied and a few previously studies (Pinruan et al. 2002, 2004a, 2004b, 2007, 2008, 2010a, 2010b, 2014; Pinnoi et al. 2003a, 2003b, 2004, 2006, 2009, 2010; Voglmayr and Yule 2006; Sivichai and Boonyuen 2010; Boonyuen et al. 2012) show a high fungal diversity in this habitat, especially in Thailand, but some of the previous studies (Pinruan et al. 2002, 2007, 2014; Pinnoi et al. 2003a, 2003b, 2004, 2006, 2009; Sivichai and Boonyuen 2010) lack molecular data. As only morphological data are insufficient to identify a fungal species (Chethana et al. 2021; Maharachchikumbura et al. 2021), studying the fungal diversity by combining morphological and molecular data are required and this has been followed in this study.

Except for *Distoseptisporahyalina* J. Yang & K.D. Hyde and *D.licualae* Konta & K.D. Hyde, most *Distoseptispora* species have been recorded as having an asexual morph and their characters, such as size, shape, colour and the number of septa in conidiophores and conidia, are crucial for distinguishing species. Morphologically, *Distoseptispora* is similar to *Ellisembia* Subram and *Sporidesmium* Link; therefore, it is problematic to recognise *Distoseptispora* species by only morphological signatures (Su et al. 2016; Hyde et al. 2019; Luo et al. 2019; Yang et al. 2021). Different studies have explored the taxonomy of *Distoseptispora* using various combinations of gene regions, such as combined ITS, LSU (Tibpromma et al. 2018), combined LSU, ITS, *rpb2* (Monkai et al. 2020) or combined LSU, ITS, *tef1*-*α* and *rpb2* (Zhang et al. 2022). In our study, we constructed the phylogenetic tree using concatenated ITS, LSU, *rpb2* and *tef1-α*. In this study, *Distoseptisporaclematidis* (MFLUCC 17-2145) and *D.nabanheensis* Jing W. Liu, X.G. Zhang & Jian Ma (HJAUP C2003) formed a sister clade, consistent with previous research (Liu et al. 2023). However, *D.clematidis* (KUN-HKAS:112708) appeared separated from these two taxa, presenting an unresolved relationship. The phylogenetic relationship amongst these three taxa is not comparable with the previous studies due to the lack of all these taxa together in their phylogenetic trees (Afshari et al. 2023; Liu et al. 2023). The unresolved clade’s origin may stem from the lack of *rpb2* sequence data for *D.clematidis* (KUN HKAS:112708) in contrast to the other two taxa where this gene region is available. This suggests that different taxon sampling and protein-coding sequences can influence the topology of the tree. However, further studies are essential to validate this hypothesis.

Morphologically, some taxa that share similarities exhibit distinct phylogenies. For instance, *D.arecacearum* shares a morphological resemblance with *D.dehongensis*, although they are phylogenetically distinct. Similarly, *D.eleiodoxae* shows morphological similarities to *D.tropica*, but resides in a separate clade in the phylogenetic tree. *Distoseptisporanarathiwatensis* forms a sister clade with *D.saprophytica* despite the differences highlighted by the pairwise comparison of ITS, LSU and other genetic markers. These encompass 22/580 bp (3.8%) and 16/870 bp (1.8%) differences for ITS and LSU, respectively, excluding gaps. Moreover, distinctions in the morphology of conidiophores and the absence of acrogenous conidia further contribute to the differentiation between *D.narathiwatensis* and *D.saprophytica*. Our study confirmed the necessity of associating molecular data with morphological characters to distinguish them, as previously demonstrated in other studies (Su et al. 2016; Hyde et al. 2019; Luo et al. 2019; Yang et al. 2021; Ma et al. 2022).

To date, the majority of *Distoseptispora* species have been reported from China (42 species) and Thailand (23 species), primarily on dead plant materials in freshwater (44 species) and terrestrial (21 species) habitats. In most cases, the hosts are unknown. Although in 19 cases, their hosts have been identified, two of which have been reported from palm, including *D.palmarum* from *Cocosnucifera* and *D.licualae* from dead leaves of *Licualaglabra* in terrestrial habitats (Hyde et al. 2016, 2021; Su et al. 2016; Xia et al. 2017; Tibpromma et al. 2018; Yang et al. 2018; Luo et al. 2019; Phookamsak et al. 2019; Monkai et al. 2020; Phukhamsakda et al. 2020, 2022; Song et al. 2020; Sun et al. 2020; Dong et al. 2021; Li et al. 2021; Shen et al. 2021; Jayawardena et al. 2022; Ma et al. 2022; Zhai et al. 2022; Zhang et al. 2022; Konta et al. 2023; Liu et al. 2023). *Distoseptispora* species have been recorded as saprophytes and their reported limited geographic distribution (China and Thailand) is most likely due to increased attention by mycologists in these areas on saprophytic fungi in aquatic and terrestrial habitats. This study shows that there is much to be done in this regard. Ongoing and future investigations will reveal the diversity and functions of these microorganisms in this ecosystem.

## Supplementary Material

XML Treatment for Distoseptispora arecacearum

XML Treatment for Distoseptispora eleiodoxae

XML Treatment for Distoseptispora narathiwatensis

## ﻿Additional information

### Author ORCIDs

Omid Karimi https://orcid.org/0000-0001-9652-2222

K.W. Thilini Chethana https://orcid.org/0000-0002-5816-9269

Antonio R.G. de Farias https://orcid.org/0000-0003-4768-1547

Raheleh Asghari https://orcid.org/0009-0006-4897-5327

Saithong Kaewchai https://orcid.org/0009-0004-9335-7697

Kevin D. Hyde https://orcid.org/0000-0002-2191-0762

Qirui Li https://orcid.org/0000-0001-8735-2890

### Data availability

All of the data that support the findings of this study are available in the main text.
